# Tobacco Smoking, Lung Cancer, and Therapy in Iraq: Current Perspective

**DOI:** 10.3389/fpubh.2018.00311

**Published:** 2018-10-26

**Authors:** Buthainah A. Ibrahim, Saif Al-Humaish, Mohammed A. I. Al-Obaide

**Affiliations:** ^1^Department of Physics, Diyala University, Baquba, Iraq; ^2^Biomedica, LLC, Sterling Heights, MI, United States; ^3^Texas Tech University Health Sciences Center, Amarillo, TX, United States

**Keywords:** tobacco smoking, incidence, health risks, cancer, proton therapy, photon therapy

## Abstract

Tobacco smoking is a research topic of high interest to the public health in Iraq. Although Iraq is a country with a high percentage of smokers, we noticed the dearth of adequate studies and programs to deal with this problem. The percentage of smokers exceed 30% of the population and smoking problem becomes a permanent habit in adults and young people. The problems associated with tobacco smoking behavior related to individuals' post-traumatic stress disorder following post-war conflicts, and the social and cultural environment. The health consequences of tobacco smoking can harm almost every organ in the body, and there are reports confirmed the tobacco smoking is a high-risk factor for lung cancer and other diseases. The relative risk of lung cancer increases with increasing duration and intensity of smoking. Also, smoking associated with bladder, prostate, and head and neck cancers, in addition to respiratory diseases. Intervention efforts should focus on reducing the prevalence of cigarette smoking, introduce effective treatments for cancer and quit smoking. In this perspective article, we present our viewpoint and three scenarios to deal with the problem of tobacco smoking in Iraq. We recommend introducing educational, health and legislative policies for quitting smoking and using effective treatments for cancer.

## Introduction

Although smoking has become a permanent habit for adults and young people in Iraq, few studies dealt with this problem. The problem of tobacco smoking in Iraq associated with the social and cultural environment, which encourages smoking and influence an individual's attitude toward cigarette smoking (1, 2). Thus, the better understanding of the uniqueness of socio-demographic characteristics of the society could help to deal more effectively with tobacco smoking problem (3–8). Another issue is the tobacco smoking negative impact on health. Cigarette smoke contains more than 4,800 chemicals, at least 69 associated with cancer (9, 10). Tobacco smoking is responsible for the majority of lung cancer cases. Also, cigarette smoking is the cause of approximately 90% of lung cancer deaths (11, 12). Another study reported that lung cancer killed approximately 1,590,000 persons in 2012 (13). Thus, lung cancer is the leading cause of cancer death, which can be avoided by quitting smoking. Also, tobacco smoking increases the risk of bladder cancer (14), head and neck cancer (15, 16) and prostate cancer death (17, 18). In addition to about 85% deaths from the chronic obstructive pulmonary disease (19, 20). This perspective article will focus on the causes of the problem in Iraq and the requirement of efficient therapies of cancer-related to tobacco smoking and measures to stop smoking.

## Smoking prevalence in iraq

The reasons for the prevalence of smoking in Iraq are individuals' behavior and the social and cultural environment that promote smoking (1, 2). The most likely cause is psychological and associated with post-war conflicts over the last three decades. A study on students in the School of Medicine in Iraq showed the reasons for smoking behavior were stress, sadness, depression, and anxiety (21). These data supported by another study performed in China, which showed that the primary reasons for smoking among medical students were stress curiosity and loneliness (22). Intriguingly, the status of parents' marriage, educational level, economic condition, and income are other influential predictive factors related to the prevalence of tobacco smoking (6, 7). Furthermore, it is possible to suggest that tobacco smoking usually acquired from parents. A study showed the importance of family influence on the smoking behavior of their teenagers (7). The decision to initiate cigarette smoking by adolescents at a young age is the outcome of transitional conflicts of normal developmental progression (8). It is unfortunate that the prevalence of smoking in Iraqi families encourages the teenagers to smoke.

Two recent studies showed that 29–31% males and 3–4% females of Iraq population are active smokers (23, 24). Although the reported percentages of cigarette smoking in Iraq population is not the highest in the Arab countries, we believe the incidence is higher than the reported. This hypothesis comes from the fact the Iraq culture does not allow women to smoke in public. Our assumption supported by a recent study carried out by Hussain and Sullivan, who stated: “it is not culturally acceptable for women to smoke, which is reflected in the prevalence rates” (24). Furthermore, Maziak et al. (23) suggested that although the incidence of tobacco smoking among Arab men is high compared to women, it is increasing among women.

## Diseases associated with tobacco smoking

Another issue related to the negative influence of smoking on health. Many Iraqi smokers show a little thought to the health risk of smoking (25). Intriguingly, most smokers know that smoking causes lung cancer. But, there is a low awareness of other health effects of tobacco. For example, smoking is a risk factor for stroke, impotence in male smokers, stained teeth, and lung cancer. These risks can harm non-smokers from secondhand smoking (26). Few studies carried out in Iraq investigated the link between tobacco smoking and cancer. A study performed during a 3 years period (2005-2007) on newly diagnosed Iraqi cancer patients registered by the Iraqi Ministry of Health showed lung cancer was the second most common cancer accounting for 8.43% of all cases of cancer, and the first most common site in males (27). Furthermore, data on lung cancer in males obtained from the annual book published by Directorate of Health in Northern Province Ninawa/Mosul/Iraq between 2000 and 2010, showed the incidence is highest (19%) in the age group 60–69 years (28). Both studies showed no data on the incidence of smokers among patients. Other studies carried out in two southern provinces Misan and Basrah, showed a higher incidence of lung cancer correlated with tobacco smoking. A study conducted in Missan province/Iraq from October 2015 to April 2016 showed the prevalence of lung cancer was 21.4%, the highest, 50.42%, in the age group 60–80 years (29). The study also showed the male: female ratio was 4:1 in the studied lung cancer patients and the percentage of tobacco smokers in the studied lung cancer patients was 78.15 %. A registry-based retrospective study in Basrah, Iraq carried out between 2005 and 2012 showed lung cancer was more than a threefold in males compared to females (30). It is possible to extrapolate from the incidence of lung cancer in males and females reported by Alhelfi (29) and Habib et al. (30) to calculate the approximate incidence of tobacco smoking among women in Iraq. The inferred incidence is higher than the reported by Maziak et al. (23) and Hussain and Sullivan (24). Thus, it is possible to suggest more than 10% of women are active smokers in Iraq. The analysis considered the established evidence that 90 out of 100 deaths from lung cancer caused by tobacco smoking (11, 12). The analysis considered the data in Iraqi lung cancer patients reported by Alhelfi (29) and Habib et al. (30). The risks of tobacco smoking on health necessitate more studies to highlight the extent of the problem and to pay attention to this problem, which takes the lives of thousands of people.

## Effective therapy of cancer

**Figure 1:**
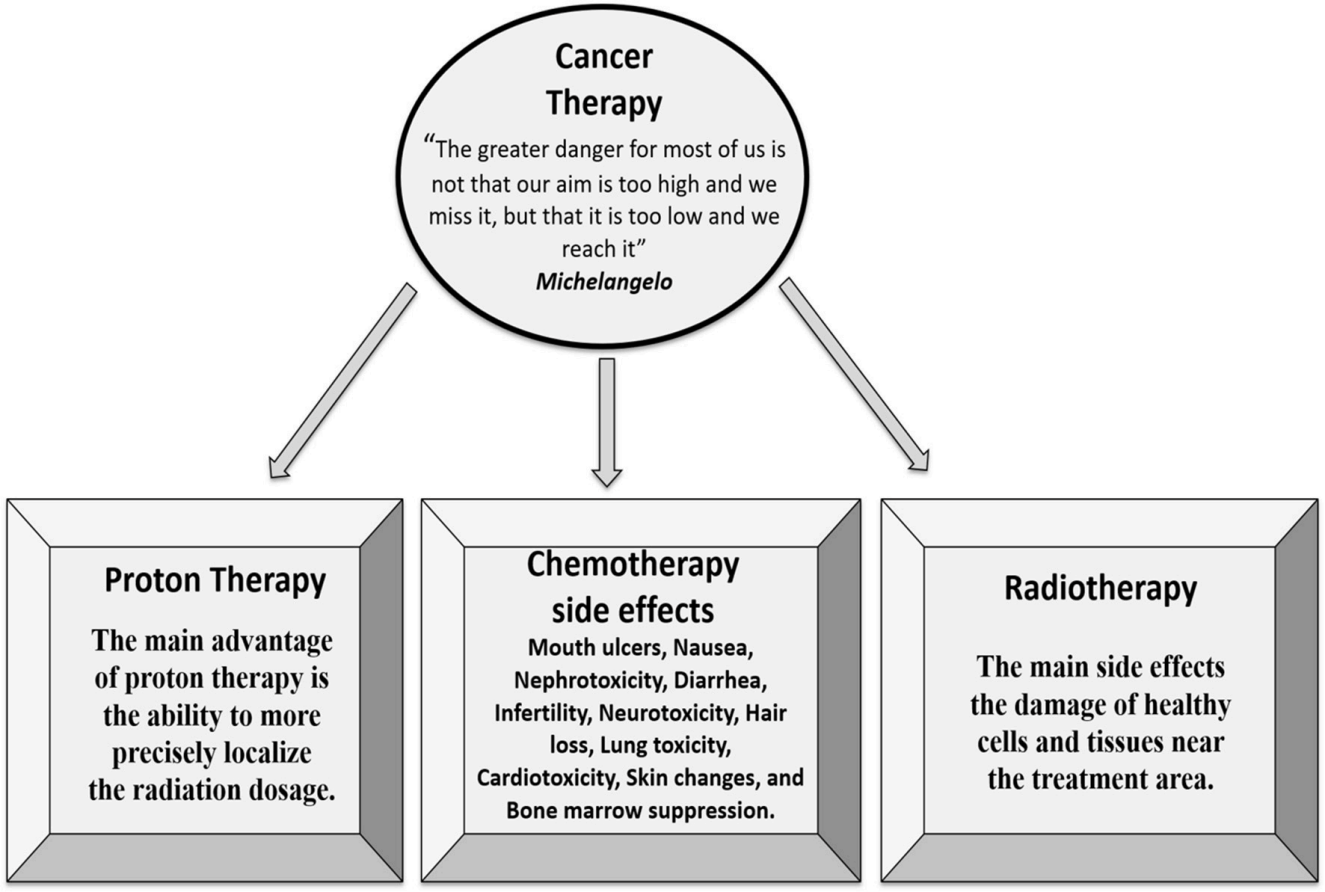
There are three main approaches to cancer therapy. The proton therapy is more efficient with fewer side effects compared with chemotherapy and radiotherapy.

Effective therapy for cancer is a problem that concerns many cancer patients in Iraq. It is a prerequisite to introducing more efficient therapeutic approaches that can reduce side effects produced by current treatment methods. The cancer therapy in Iraq is devoid of modern treatment techniques that reduce side effects produced by current treatments. Currently, the available treatments in Iraq: radiotherapy, chemotherapy and hormone therapy, which cause severe side effects. Here we discuss a therapy currently not used in Iraq. The proton beam therapy, which may potentially mitigate treatment-related toxicities by minimizing the dose to healthy organs in the treatment. The limited range of protons has the potential to reduce normal tissue toxicity compared to photon radiotherapy (31, 32). Protons have different dosimetric characteristics than photons used in conventional radiation therapy. After a short build-up region, traditional radiation shows an exponentially decreasing energy deposition with increasing depth in tissue. In contrast, protons show an increasing energy deposition with penetration distance leading to a maximum (the “Bragg peak”) near the end of the range of the proton beam. As protons charged particles, a pencil proton beam can accurately guided toward the tumor. Because protons are massive particles, they penetrate with the minimal prevalence, and they slow down comparatively fast when entering biological tissues. Due to their relatively large mass, protons are located and have slight transverse side dispersion in tissues; the beam is not widened frequently, stays focused on the tumor and transports low-dose to healthy tissues surrounded the tumor. All protons of specified energy have specific range; few protons penetrate behind that distance. Moreover, proton beams offer the advantage of accurate dose localization and suitable dose-depth distributions, in comparison with photon radiotherapy in which neighboring tissues to the tumor may receive a similar dose and can be damaged (33). Thus, proton therapy compared to photon therapy has the advantage of the localization of treatment in depth but implies higher costs for the accelerator and beam lines (34). There is a growing interest in the use of proton therapy for the treatment of many cancers, for example, the therapeutic efficacy of prostate proton radiation allows for an increase in dose without a substantial increase in side effects (35, 36). We suggest introducing proton therapy for the treatment of cancers in Iraq. The suggested treatment is more effective and without substantial side effects. However, the significant cost of proton beam therapy remains a barrier to its common usage (37). In Figure 1, we summarized the advantages and disadvantages of main cancer therapy technologies.

## Programs to encourage quitting smoking

Programs for the encouragement to quit tobacco smoking is central in the drive to reduce chronic disease due to tobacco smoking. Comprehensive and active awareness programs are the primary tools for smoking prevention and cessation (23). The mass media play a significant role to support the efforts to control this habit through the explanation of the risks of smoking (38). Other activities in this direction include endorsing tobacco smoking free public places, group behavior therapy, and support to the family smoking control programs to reduce children's exposure to environmental tobacco smoke (39–41). A study provided to the Ministry of Health Promotion (MHP) of Canada summarized the effective and cost-effective in the short-term smoking cessation strategies based on pharmacotherapy of tobacco smoking, quitting smoking, hospital-based interventions, and telephone counseling (42). Quitting smoking is the wish of most U.S. adult cigarette smokers, 68.0% (43). Whereas, 45.5% of all U.S. high school students attempted to stop smoking (44). Most smokers can quit without using treatments (43). However, treatments are available and shown to be useful for smokers who want to quit smoking. Examples of treatments proven to be effective to quit smoking: nicotine replacement products include: nicotine patch, inhaler, nasal spray; and non-nicotine medications: bupropion SR and varenicline tartrate (45, 46).

## Post-conflict scenarios to quit smoking in iraq

Here, we postulated sequence events to develop scenarios to quit smoking. The smoking problem in Iraq is part of the psychological problem associated with postwar conflicts continued since 1980 A.D. Conflicts persisted over three decades can cause the Post-traumatic Stress Disorder (24, 47, 48). Although Iraq is one of the WHO Framework Convention on Tobacco Control (FCTC) 188 signatories, so far smoking cessation programs and tobacco prevention measures had almost no traction (24). As a result, the people of Iraq will continue to suffer an increased risk of developing smoking-related diseases. The proposed scenarios are to encourage smokers to participate in a program for post-traumatic stress disorder treatment to stop smoking and to initiate successful tobacco control policies of other post-conflict countries that have similar cultures and conflicts, for example, Pakistan and Iran (24). The problem requires collaboration between health, education and legislative sectors to show to the People of Iraq the health benefits following stopping smoking. Briefly, we propose three scenarios for the post-conflict program to quit smoking in Iraq. First scenario: Introducing two curricula by the Ministry of Education and Ministry of Higher Education and Scientific Research for adolescents' students in the 9th grade secondary school and the 1st year University respectively. The curricula include the updated data on the health risk and diseases caused by tobacco smoking (49–51) and the harm caused to their health and health of non-smoking people (52–54). Second scenario: Health sector can provide reasonable Post-traumatic Stress Disorder Treatment Clinic(s) for smokers who suffer frightening, stressful, and distressing life events and are willing to quit cigarette smoking. Also, we recommend treatments to quit smoking and for effective treatments for cancer. Third scenario: Follow-up to enforce the legislative laws to have a culture that understands the health risks linked to tobacco smoking and to endorse the Smoke-Free Places Act. Together, the three scenarios will provide effective policies for reducing smoking prevalence in Iraq by preventing non-smokers from initiating smoking and encouraging current smokers to quit.

## Conclusions

In Iraq, Tobacco smoking is an epidemic problem. The focus of this study was on the prevalence and causes of tobacco smoking and the negative impact on the health of the People of Iraq. The most important cause of smoking prevalence related to the social, cultural setting and posttraumatic stress disorder that promote the smoking habit. To handle the problem, we suggested three scenarios to promote smoking cessation: launching anti-smoking curricula for adolescents, reasonable health care for Posttraumatic Stress Disorder Treatment and effective treatments for cancer and quitting smoking and enforcing legislative laws to reduce the health risks and the consequences of tobacco smoking on the smokers and the society.

## Author contributions

All authors listed have made a substantial, direct and intellectual contribution to the work, and approved it for publication.

### Conflict of interest statement

The authors declare that the research was conducted in the absence of any commercial or financial relationships that could be construed as a potential conflict of interest.
